# Editorial: 50th Anniversary of Adult Neurogenesis: Olfaction, Hippocampus, and Beyond

**DOI:** 10.3389/fnins.2016.00319

**Published:** 2016-06-30

**Authors:** Alino Martinez-Marcos, José L. Trejo, Laura López-Mascaraque

**Affiliations:** ^1^Neuroplasticity and Neurodegeneration Laboratory, CRIB, Ciudad Real Medical School, Universidad de Castilla-La ManchaCiudad Real, Spain; ^2^Laboratory of Adult Neurogenesis, Department of Molecular, Cellular and Developmental Neurobiology, Cajal Institute, Consejo Superior de Investigaciones CientíficasMadrid, Spain; ^3^Laboratory of Olfactory System Development, Department of Molecular, Cellular and Developmental Neurobiology, Cajal Institute, Consejo Superior de Investigaciones CientíficasMadrid, Spain

**Keywords:** Alzheimer, dopamine, glia, epilepsy, exercise, stroke

In the mid-sixties a novel discovery faced the traditional idea on the immutability of the adult brain. Up to then, scientist assumed that once the brain has reached its maturity neurons can die, but nor regenerate—e.g., “*Once the development was ended, the founts of growth and regeneration of the axons and dendrites dried up irrevocably. In the adult centers, the nerve paths are something fixed, ended, and immutable. Everything may die, nothing may be regenerated. It is for the science of the future to change, if possible, this harsh decree*” (Cajal, 1914). In 1965, Altman and Das published their seminal article (Altman and Das, 1965), although Altman had already suggested this idea years earlier (Altman, 1962, 1963). This discovery was neglected up to 80's when Fernando Nottebohm demonstrated adult neurogenesis in the avian brain related (Nottebohm, 1981). In the following decade, two main niches of adulthood neurogenesis were characterized in the mammalian brain: the subventricular zone of the lateral ventricle from where neuroblasts migrate to the olfactory bulb and the subgranular layer of the dentate gyrus for turnover of hippocampal granule cells.

Since then, a number of experimental data have tried to unravel the role of bulbar and hippocampal newly-born neurons. Among others, adult neurogenesis has been related to learning and memory, but its exact function in the pre-existing circuits is far from clear and the relevance of glial-neuronal interactions has been only envisaged. It has been demonstrated that neurogenic rate and morphology of adult-born neurons can be regulated by external factors such as sensory stimuli, exercise, -sexual- experience, and stress through given molecular pathways. This rate can be altered during disease, particularly in stroke, epilepsy Down syndrome and neurodegenerative disorders, and its potential therapeutic capacity is being investigated even though this neurogenic capacity still needs to be further explored in human brain.

This Research Topic addresses half-century advances on all these topics from a multidisciplinary point-of-view. We suggest readers to begin with the editorial of a related Research Topic by Peretto and Bonfanti, to follow with a historical view (Bonfanti), and a number of articles from worldwide leaders in the field. Vicario-Abejón's group (Nieto-Estevez et al.) has reviewed the action of IGF-I signaling in a variety of *in vitro* and *in vivo* models, focusing on the maintenance and proliferation of NSCs/progenitors, neurogenesis, and neuron integration in synaptic circuits. Mira's laboratory (Vilar and Mira) focused on the current understanding of neurotrophin modulation of adult neurogenesis, identifying both expected and unexpected functions for the neurotrophin protein family of ligands. De Marchis' group (Bonzano et al.) reviewed the emerging aspects related to dopaminergic cells heterogeneity, molecular determinants of adult born dopaminergic neurons, their plasticity and function in the olfactory bulb. Kohl's laboratory (Salvi et al.) provides evidence that species and the specific strain largely matter when investigating effects of the generation of new neurons in neurogenic and non-neurogenic regions following dopamine agonists treatment. Avila's group (Llorens-Martin et al.) has focused their review on the morphological alterations of the dendritic tree of newborn neurons both in the physiological process and in neurodegeneration, while Tsuboi's laboratory (Yoshihara et al.) reviewed the molecular mechanisms that underlie the sensory input-dependent development of newborn interneurons and the formation of functional neural circuitry in the olfactory bulb.

López-Mascaraque's team (Figueres and López-Mascaraque) addressed the distribution and neurochemical identity of adult olfactory bulb interneurons targeted at either embryonic or postnatal ages with a ubiquitously expressed transposable reporter vectors encoding eGFP. Raineteau's group (Azim et al.) discusses the role of a strict spatial coding of segregated NSCs populations during oligodendrogenesis. Kuo's laboratory (Adlaf et al.) emphasizes the relevance of how neural circuit-level input can be a distinct characteristic defining postnatal/adult NSCs from non-neurogenic astroglia. Ortega's group (Ortega and Costa) reviewed the state-of-the-art of live imaging as well as the alternative models that currently offer new answers to critical questions. Saghatelyan's laboratory (Gengatharan et al.) analyzes the pivotal role of astroglial cells in adult neurogenesis. Suarez's group (Pérez-Martín et al.) suggested a potential modulatory role for PPARalpha in the age-induced neurogenesis decline. Paredes' laboratory demonstrated that while mating behavior influences the process of olfactory bulb neurogenesis (Corona et al.), sexual behavior induces long-lasting plastic changes in the olfactory bulb (Unda et al.).

Malgrange's group (Marlier et al.) reviewed stroke-induced adult neurogenesis, from a cellular and molecular perspective, to its impact on brain repair and functional recovering. Parent's laboratory (Korn et al.) reveals the Importance of adult neurogenesis in maintaining network stability and suggesting that this circuit is a potential target for anti-epileptogenic interventions. Encinas' team (Pineda and Encinas) discusses the mechanisms by which neuronal hyperexcitation influences hippocampal neurogenesis. Varea's group (López-Hidalgo et al.) shows a reduction in the number of proliferating cells in trisomic mice, although the final number of neurons integrated in the system is the same in Ts65Dn, a Down syndrome mice model. Lazarov's laboratory (Hollands et al.) discussed the association between impairments in adult hippocampal neurogenesis and cognitive deficits leading to Alzheimer's disease. Martínez-Marcos team (De la Rosa-Prieto et al.) characterizes the neurogenic process in the olfactory bulb of APP/PS1 mice analyzing the neurogenic and neurodegenerative rates of new and preexisting interneuron populations. Finally, Trejo's team (Gradari et al.) hypothesizes on adult neurogenesis as a physical substrate for hormetic, biphasic dose-responses to exercise on cognition and mood.

Fifty years after the birth of adult neurogenesis, the health of this field is very good, as demonstrated by this Research Topic, covering hot aspects of this area of Neuroscience. The data emerging from these 22 contributions addressed issues of fundamental importance for understanding how neural cells could be integrated into existing functional brain circuits. Furthermore, these contributions point to the fact that much more knowledge is still needed in basic features of adult neural cell genesis. The number of unexplored aspects of this field appears to be so measureless that we conclude that this is a healthy 50-years-old baby field. Hopefully, at the 75th anniversary, our field will be mature enough to cover the translational edge of this topic.

## Author contributions

AM, JT, and LL have written this editorial for the Research Topic they have edited.

### Conflict of interest statement

The authors declare that the research was conducted in the absence of any commercial or financial relationships that could be construed as a potential conflict of interest.
